# A Case of Eosinophilic Granulomatosis With Polyangiitis Presenting With Conjunctival Lesions During Treatment With IL‐5 Inhibitors

**DOI:** 10.1155/crrh/9989981

**Published:** 2026-10-07

**Authors:** Shin-Ichiro Ohmura, Soma Fukami, Kazuhisa Uemura, Yoshiro Otsuki

**Affiliations:** ^1^ Department of Rheumatology, Seirei Hamamatsu General Hospital, 2-12-12 Sumiyoshi Chuo-ku Hamamatsu, Shizuoka 430–8558, Japan, seirei.or.jp; ^2^ Department of Oculoplastic and Orbital Surgery, Seirei Hamamatsu General Hospital, 2-12-12 Sumiyoshi Chuo-ku Hamamatsu, Shizuoka 430–8558, Japan, seirei.or.jp; ^3^ Department of Pathology, Seirei Hamamatsu General Hospital, 2-12-12 Sumiyoshi Chuo-ku Hamamatsu, Shizuoka, Japan, seirei.or.jp

**Keywords:** conjunctival nodules, eosinophilic granulomatosis with polyangiitis, mepolizumab

## Abstract

Eosinophilic granulomatosis with polyangiitis (EGPA) is a systemic rheumatic disease characterized by necrotizing vasculitis in the small vessels with eosinophilic inflammation. EGPA involves multiple organs, such as the heart, lungs, kidneys, gastrointestinal tract, nerves, and ophthalmic organs. Ophthalmic involvement includes conjunctival nodules, orbital myositis, orbital inflammatory syndrome, dacryoadenitis, and cranial nerve palsy. Notably, conjunctival nodules in patients with EGPA are very rare lesions but may present with atypical eyelid swelling. Herein, we present a case of EGPA with bilateral conjunctival nodules that developed during treatment with Interleukin (IL)‐5 inhibitors. This case highlights that EGPA may exhibit bilateral conjunctival lesions even during treatment with IL‐5 inhibitors, suggesting incomplete suppression of *T* helper Type 2–associated immune pathways.

## 1. Introduction

Eosinophilic granulomatosis with polyangiitis (EGPA) is a systemic rheumatic disease characterized by eosinophilic inflammation and necrotizing vasculitis of small‐to‐medium‐sized vessels [1]. Recent advances in biologics targeting the Interleukin (IL)‐5 axis have substantially improved disease control in EGPA by effectively suppressing peripheral eosinophilia. However, these therapies may also modify the clinical manifestations and relapse patterns of EGPA, making recognition of localized disease activity increasingly important [2].

Ophthalmic involvement is uncommon in EGPA and includes conjunctival nodules, orbital myositis, orbital inflammatory syndrome, dacryoadenitis, and cranial nerve palsy [3–5]. Previous reports have described conjunctival lesions as inflammatory or granulomatous masses presenting with eyelid swelling rather than typical necrotizing granulomas [4, 5]. To our knowledge, however, bilateral conjunctival nodules developing during ongoing IL‐5‐targeted biologic therapy have not been previously reported.

Herein, we report a patient with EGPA who developed bilateral conjunctival nodules during treatment with IL‐5‐targeted biologics.

## 2. Case Presentation

A 58‐year‐old man presented with bilateral eyelid swelling. He had intractable bronchial asthma (BA) for 40 years and frequently required adjunctive glucocorticoids. Twenty‐six months before admission, he developed weight loss, cough, and purpura (Supporting Figure 1) but did not have neuropathy. His blood eosinophil count was 10,630/μL with increasing C‐reactive protein (CRP) of 1.68 mg/dL. Whole‐body computed tomography (CT) revealed sinusitis, and multiple pulmonary infiltrates in the lungs but showed no specific lesions suggestive of IgG4‐related disease (RD) (Figure 1). A skin biopsy specimen obtained from a purpuric lesion around the right lateral malleolus demonstrated vasculitis without eosinophilic infiltration or extravascular granulomas (Supporting Figure 2a,b). At the initial diagnosis, ANCA was negative and serum IgG4 was not measured. He was diagnosed with EGPA based on the 2022 American College of Rheumatology (ACR)/European Alliance of Associations for Rheumatology (EULAR) classification criteria for EGPA [1]. The Birmingham Vasculitis Activity Score (BVAS) was 12. The revised Five‐Factor Score (FFS) was 0 at the initial diagnosis [6]. At the initial diagnosis of EGPA, the patient had no ocular manifestations, including eyelid swelling. Therefore, orbital imaging was not performed. The patient was treated with high‐dose prednisolone (PSL) for the skin and pulmonary lesions, but his BA was intractable, and benralizumab (30 mg every 2 months) was initiated. However, 15 months after initiation of benralizumab, scleritis developed without relapse of other EGPA manifestations or peripheral eosinophilia. Eyelid swelling subsequently developed. Thus, the PSL dose was increased from 8 to 40 mg, and benralizumab was switched to mepolizumab 300 mg every month due to intractable scleritis and eyelid swelling. However, eyelid swelling relapsed when the PSL dose was 10 mg (Figure 2). Therefore, he was admitted to our hospital for surgical treatment.

**FIGURE 1:**
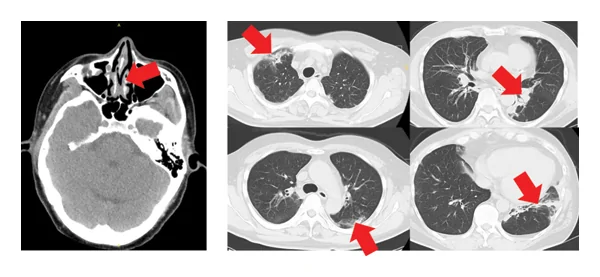
Computed tomography showing sinusitis and multiple infiltrative shadows in the lungs.

**FIGURE 2:**
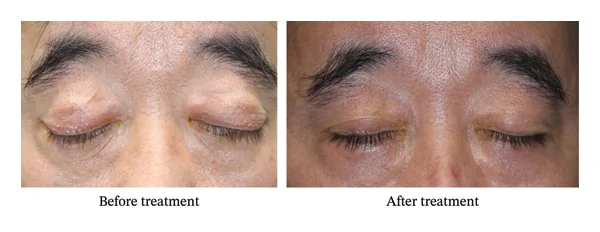
Eyelid swelling before and after surgical treatment.

On admission, his body temperature was 36.8°C, and his body weight had not decreased. Physical examination revealed no abnormal findings except for bilateral eyelid swelling and bilateral conjunctival nodules (Figure 3). Blood test results were as follows: white blood cell count, 10,000/μL; neutrophil count, 8160/μL; lymphocyte count, 1230/μL; eosinophil count, 20/μL; total protein, 7.2 g/dL; albumin, 4.5 g/dL; aspartate aminotransferase, 20 U/L; alanine aminotransferase, 35 U/L; lactate dehydrogenase, 234  U/L; creatine kinase, 84  U/L; blood urea nitrogen, 16 mg/dL; creatinine, 0.94 mg/dL; CRP, 0.06 mg/dL; CH50, 61 U/mL; C3, 138 mg/dL; C4, 29 mg/dL; immunoglobulin *E* (IgE), 115.0 U/mL; IgG, 773 mg/dL; IgG4, 28.1 mg/dL; soluble IL‐2 receptor, 291 U/mL; angiotensin‐converting enzyme, 7.7 U/L; B‐D glucan, < 5.0 pg/mL; aspergillus antigen, negative; ANA < 40; anti‐SS‐A antibody negative; and myeloperoxidase (MPO)‐antineutrophil cytoplasmic antibody (ANCA) and Proteinase 3 (PR3)‐ANCA were negative. The urinalysis revealed no abnormalities. No clinical or laboratory findings suggestive of infection were observed at admission. Therefore, the neutrophilic predominance was considered to be attributable to glucocorticoid therapy. Whole‐body CT did not reveal any abnormal findings, including IgG4‐RD‐specific lesions, such as the pancreas, retroperitoneum, and kidneys. Magnetic resonance imaging revealed masses on both upper eyelids (Supporting Figure 3). Histopathological examination of the conjunctival nodule revealed dense inflammatory cell infiltration composed of lymphocytes, plasma cells, and eosinophils. Immunohistochemistry demonstrated 127 IgG4‐positive plasma cells per high‐power field, with an IgG4/IgG‐positive cell ratio of 71.8%. Neither storiform fibrosis nor obliterative phlebitis was identified. In addition, no necrotizing vasculitis or extravascular granulomas were observed. The pathological findings were therefore characterized predominantly by eosinophilic and lymphoplasmacytic inflammation (Figure 4a,b). His total score for the 2019 ACR/EULAR classification criteria for IgG4‐RD was 20 (histopathological pattern [+4], IgG4 immunostaining [+16]) [7]. However, the presence of marked eosinophilia (> 3000/μL) at disease onset, combined with the absence of IgG4‐RD‐specific histopathological features—such as storiform fibrosis and obliterative phlebitis—resulted in no findings suggestive of IgG4‐RD [7]. The patient’s BVAS was two points, and the revised FFS was 1. After surgical treatment, the patient’s condition improved, and eyelid swelling did not relapse (Figure 2). At the last follow‐up, the patient was treated with PSL 6 mg, methotrexate 10 mg/week, and mepolizumab 300 mg/month.

**FIGURE 3:**
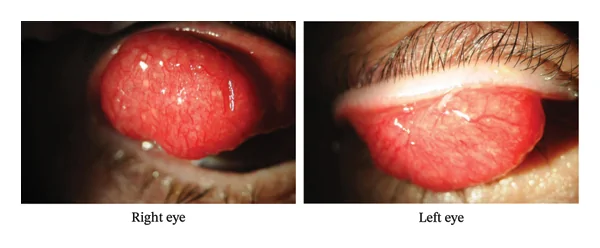
Patient showing bilateral conjunctival nodules.

**FIGURE 4:**
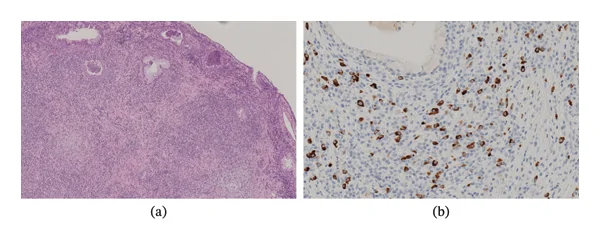
(a) Hematoxylin and eosin staining of his conjunctival nodule (× 4). Pathological findings showing inflammatory cell infiltration consisting of lymphocytes, plasma cells, and eosinophils without storiform fibrosis and obliterative phlebitis. (b) IgG4 immunostaining in the patient’s conjunctival nodule (× 20). Immunohistochemical staining for IgG4. IgG4‐positive plasma cells (127 cells/HPF; HPF area, 0.24 mm^2^) were identified. His IgG4/IgG ratio was 71.8%.

## 3. Discussion

We report a case of EGPA that developed bilateral conjunctival nodules during treatment with IL‐5 inhibitors, which provide two clinical insights. First, bilateral conjunctival nodules developed during treatment with IL‐5 inhibitors despite normal blood eosinophil count. Second, IgG4‐RD‐like lesions were observed in conjunctival nodules.

EGPA is a systemic rheumatic disease characterized by small‐ and medium‐vessel necrotizing vasculitis and eosinophilic inflammation. EGPA is a Type 2 helper *T* (Th2) cell‐mediated disease, and upregulation of the IL‐5 and IL‐4/IL‐13 pathways are related to EGPA development [8, 9]. Th2‐related cytokines are therapeutic targets in patients with EGPA, and IL‐5 inhibitors, such as mepolizumab and benralizumab, have demonstrated excellent efficacy in severe EGPA [10, 11]. In contrast, ophthalmologic manifestations are very rare in EGPA, with a reported prevalence of 0.7% [6, 12, 13]. Most previously reported EGPA cases with ocular manifestations occurred at disease onset, whereas our case demonstrates new‐onset conjunctival nodules during IL‐5 inhibitors [12, 13]. A previous report showed that IL‐5 inhibition markedly suppresses peripheral eosinophilia, which may mask disease activity and delay recognition of relapse [14]. The present case highlights that new organ involvement, including ocular lesions, can develop despite effective peripheral eosinophil depletion.

The appearance of intractable conjunctival nodules despite complete depletion of peripheral blood eosinophils suggests a dissociation between the effects of IL‐5 inhibitors in the systemic circulation and in localized tissues. Mepolizumab and benralizumab target the IL‐5 pathway through distinct mechanisms. Mepolizumab neutralizes circulating IL‐5, whereas benralizumab binds to IL‐5 receptor *α* expressed on eosinophils and induces antibody‐dependent cell‐mediated cytotoxicity, resulting in profound depletion of circulating eosinophils. Despite these mechanistic differences, both agents primarily suppress systemic eosinophilic inflammation and may not completely eliminate localized tissue inflammation. This may explain why switching from benralizumab to mepolizumab failed to control conjunctival inflammation in our patient. A previous study reported that while mepolizumab nearly completely depletes peripheral blood eosinophils, it reduces eosinophil numbers in the airway mucosa by only 55% [14]. Another report also demonstrated that localized eosinophilic inflammation may persist despite IL‐5 inhibitors effectively reducing peripheral blood eosinophils [15]. Persistent tissue eosinophilia may also be maintained by local immune mechanisms independent of systemic IL‐5 blockade, including locally produced eotaxins and survival factors such as GM‐CSF [16]. These mediators may contribute to compartmentalized eosinophilic inflammation despite effective depletion of circulating eosinophils. These findings suggest that localized tissue environments may respond differently to systemic IL‐5 inhibition compared with the peripheral circulation. However, these data are limited, and larger prospective studies are warranted to confirm these observations.

IgG4‐RD is characterized by elevated serum IgG4 levels and marked infiltration of IgG4‐positive plasma cells in affected tissues [6]. Pathogenically, *T* helper Type 2 (Th2)‐related cytokines, such as IL‐4 and IL‐13, promote IgG4 class switching, representing a shared immunopathogenic pathway between EGPA and IgG4‐RD. Serum IgG4 levels are often significantly elevated in patients with active EGPA and have been shown to correlate with the number of organ manifestations and disease activity [17–19]. Previous studies have reported that EGPA can present with clinical features mimicking IgG4‐RD, such as IgG4‐RD‐like eyelid swelling [20–24]. Table 1 summarizes the reported cases of EGPA presenting with IgG4‐RD‐like eyelid lesions, including the present case. In our patient, IgG4‐RD‐like lesion was localized to the eyelid, with no other lesions suggestive of IgG4‐RD and no elevation in serum IgG4 levels, which was consistent with previous report [23]. These findings suggest that the prominent IgG4‐positive plasma cell infiltration observed in the conjunctival lesion may represent a secondary reactive immunological phenomenon driven by chronic Th2‐mediated inflammation rather than a true overlap between EGPA and IgG4‐RD.

**TABLE 1:** EGPA with IgG4‐like eyelid lesions.

	Case 1	Case 2
Age (years)	62	58

Sex	M	M

Allergic diseases	Asthma	Asthma
		Sinusitis

Other EGPA manifestations	Nerve	Pneumonia
	Skin	Purpura
		Scleritis

IgG4‐like lesion	Eyelid	Eyelid

Disease duration	16 months	2 years

Duration of IL‐5 treatment	Not used	2 years

Treatment at onset of eyelid swelling	PSL 10 mg	PSL 8 mg, benralizumab

CRP (mg/dL)	NA	0.06

ANCA‐status	MPO‐ANCA 237 IU/mL	Negative

Serum eosinophil count (/μL)	2260	20

Serum IgE (IU/L)	21.4	115

Serum IgG4 (mg/dL)	119	28.1

Pathological findings	Lymphoplasmacytic infiltration with lymphoid follicle formation	Inflammatory cell infiltration consisting of lymphocytes, plasma cells, and eosinophils.
	IgG4/IgG ratio 50%	Absence of storiform fibrosis and obliterative phlebitis
		127 IgG4‐positive plasma cells per high‐power field
		IgG4/IgG ratio 71.8%

Additional treatment	GC, IVCY, IVIG	Surgical treatment

Outcome	Improved	Improved

Reference	[23]	Our case

*Note:* ANCA, antineutrophil cytoplasmic antibody; EGPA, eosinophilic granulomatosis with polyangiitis; GC, glucocorticoid; IgE, immunoglobulin *E*; IgG4, immunoglobulin G4; IL, interleukin; EGPA, eosinophilic granulomatosis with polyangiitis; GC, glucocorticoid; IgE, immunoglobulin *E*; IgG4, immunoglobulin G4; IL, interleukin.

Abbreviation: CRP, C‐reactive protein.

Although concomitant IgG4‐RD cannot be completely excluded, our patient lacked characteristic organ involvement such as the salivary glands, lacrimal glands, pancreas, and retroperitoneum. Furthermore, the conjunctival biopsy showed abundant IgG4‐positive plasma cells but lacked storiform fibrosis and obliterative phlebitis, which are characteristic histopathological features of IgG4‐RD. Therefore, we considered the conjunctival lesions more likely to represent localized IgG4‐rich inflammation associated with EGPA rather than a separate manifestation of IgG4‐RD. Nevertheless, because EGPA and IgG4‐RD share Th2‐skewed immune responses and increased IgG4 production, distinguishing these entities in individual patients remains challenging.

Ocular lesions in granulomatosis with polyangiitis (GPA) are histopathologically characterized by granulomatous inflammation, often accompanied by vasculitis. In contrast, the conjunctival biopsy in our patient demonstrated predominantly eosinophilic and lymphoplasmacytic inflammation without granulomatous inflammation or necrotizing vasculitis, supporting localized eosinophilic inflammation associated with EGPA rather than GPA [25].

In the present case, the patient had isolated ocular involvement with a BVAS of 2 and no evidence of major organ‐threatening disease. Therefore, surgical excision combined with methotrexate was considered an appropriate treatment strategy. In contrast, more intensive systemic immunosuppressive therapy, such as cyclophosphamide or rituximab, may be more appropriate for patients with systemic or organ‐threatening manifestations.

Although conclusions are limited by the single‐case nature of this observation, larger prospective studies will be required to determine the frequency and clinical significance of these lesions in EGPA during IL‐5 inhibitors.

## Author Contributions

SO had full access to all data. SO was responsible for the organization and coordination of the case.

## Funding

This study did not receive any specific grants from funding agencies in the public, commercial, or nonprofit sectors.

## Disclosure

All authors approved the final version of this article.

## Consent

Signed informed consent was obtained from the patient regarding the use of patient information to write a case report.

## Conflicts of Interest

The authors declare no conflicts of interest.

## Supporting Information

Additional supporting information can be found online in the Supporting Information section.

## Supporting information


**Supporting Information** Supporting Figure 1: Patient showing purpura. Supporting Figure 2: Skin biopsy findings. (a) There is thickening of the vessel walls accompanied by perivascular lymphocytic infiltration and extravasation of erythrocytes in the superficial dermis (× 20. Hematoxylin–eosin staining). (b) Fragmentation of elastic fibers is observed in medium‐sized arteries in the deep dermis (× 20. Elastica van Gieson staining). Supporting Figure 3: Magnetic resonance imaging revealed masses in both upper eyelids.

## Data Availability

The data underlying this article will be shared upon reasonable request to the corresponding author.
